# Bennett's Fracture

**Published:** 1913-03-15

**Authors:** 


### BENNETT'S FRACTURE.

Cases of that special variety of fracture of the metacarpal bone of the thumb, to which Professor Bennett, of Dublin, first drew attention twenty-seven years ago (*British Medical Journal*, 1886), probably occur with considerable frequency. It would appear, however, that they are by no means always recognised, for the descriptions of the fracture in most of the ordinary text-books of surgery still render it necessary to refer to Professor Bennett's original monograph.

The lesion there described consists in a separation of the whole or a portion of the triangular process carrying the saddle-shaped facet for articulation with the trapezium, which is such a prominent feature of the carpal end of the first metacarpal bone. It will be remembered that this process is inclined to the ulnar aspect of the hand, and clearly indicates the side to which the bone belongs.

The fracture usually occurs in comparatively young people, and the patient complains in nearly every instance of a sprained thumb; the injury causing the so-called sprain being a fall upon the hand with the thumb in the attitude of opposition.

The prominent symptoms are moderate pain and inability to use the thumb in any way. In all cases there is swelling and tenderness over the base of the first metacarpal bone and the metacarpo-trapezial joint, but in none is there marked deformity. Crepitus can usually be obtained by combining traction on the thumb with pressure over the base of its metacarpal bone. Simple traction immediately gives rise to pain over the site of fracture. The crepitus is not of the harsh and grating character met with in most fractures; it has a softer quality reminiscent of that met with in separation of an epiphysis.

The diagnosis is easily verified by *x*-ray examination; when the exact condition of affairs is seen to be an oblique fracture running downwards and inwards through the base of the metacarpal bone, detaching the pyramidal portion which carries the major part of the facet for the trapezium. It will be obvious that the fracture is in part intracapsular, and largely concerns the articular cartilage—a fact which may explain the soft character of the crepitus above referred to.

It is of some interest to speculate upon the exact mode of production of this type of fracture. Owing to the peculiar shape of the base of the bone, it is apparent that, during opposition of the thumb, the point on the anterior aspect of the base of the metacarpal becomes firmly pressed against the trapezium, and that it is this that limits the degree to which the thumb can be opposed or adducted under normal circumstances. If, when in the position of opposition, great force be applied to the shaft of the thumb, it is clear that the pressure between the trapezium and the base of the metacarpal bone may become so great that the articular portion of the latter is pushed off, and the rest of the metacarpal bone displaced upwards on to the dorsal aspect of the trapezium. It is important to differentiate Bennett's fracture from the form of fracture of the shaft of the metacarpal bone near the base which results from a different form of violence.

